# Hierarchical Structures in Livestock Trade Networks—A Stochastic Block Model of the German Cattle Trade Network

**DOI:** 10.3389/fvets.2020.00281

**Published:** 2020-05-27

**Authors:** Laura Brzoska, Mareike Fischer, Hartmut H. K. Lentz

**Affiliations:** ^1^Institute of Mathematics and Computer Science, University of Greifswald, Greifswald, Germany; ^2^Institute of Epidemiology, Friedrich-Loeffler-Institut, Greifswald-Insel Riems, Greifswald, Germany

**Keywords:** network analysis, epidemic model, cattle trade, Germany, modularity, stochastic block model

## Abstract

Trade of cattle between farms forms a complex trade network. We investigate partitions of this network for cattle trade in Germany. These partitions are groups of farms with similar properties and they are inferred directly from the trade pattern between farms. We make use of a rather new method known as stochastic block modeling (SBM) in order to divide the network into smaller units. SBM turns out to outperform the more established community detection method in the context of disease control in terms of trade restriction. Moreover, SBM is also superior to geographical based trade restrictions and could be a promising approach for disease control.

## 1. Introduction

The trade with living animals poses a major risk for the spread of infectious diseases. The latter include foot-and-mouth disease (1–3) and bovine virus diarrhea (4, 5), as well as zoonotic diseases, such as bovine tuberculosis (6, 7). Cattle farmers typically sell and/or purchase animals at a relatively high frequency and to different trading partners. Therefore, the trade between all involved farmers forms a complex network, where in case of an outbreak many farms can be infected within a short period of time.

In order to understand the structure of these trade connections as well as to quantify the risk of infection spread, the trade data can be represented as a complex network that can be analyzed mathematically (8, 9). Concerning trade data, all EU member states are obliged to report any cattle movement to a central database (10). The usage of this data is, however, typically restricted to competent authorities. Once the data is available, common network analyses focus on ranking the involved farms – nodes in the network with edges, i.e., trade connections, between them – according to their suitability for disease containment and surveillance. Highly ranked farms are then called central nodes. It has been shown that node rankings can be helpful for efficiently implementing countermeasures such as targeted vaccination (11–15). The second common goal of network analyses is understanding the large scale structure of the studied system. Typically, livestock trade networks in developed countries consist of up to 10^5^ farms (15–19). Therefore, finding inherent structures that allow to partition a network into small subsets of nodes that are in the best case independent from each other, is a promising way to gain an understanding of the system as a whole. In addition, partitioning the network has another advantage: epidemics can be fought considerably better in systems consisting of smaller units. Moreover, a trade network could be constructed out of many small independent subunits on purpose. This is known as compartmentalization and can be considered as a method for passive disease protection.

The simplest example of such a partitioning is the component structure. It determines which pairs of farms can potentially infect each other at all via trade, directly or indirectly. In other words, the component structure describes whether the network consists of a large continent or a number of small disconnected islands. The component structure of a large network such as livestock trade typically yields very large structures that can be used to assign nodes to two disjoint risk classes (13, 15, 20–23). First, nodes that can reach a large number of other nodes through trade and second, nodes that can only reach a small number of others through trade.

Although partitioning nodes according to the component structure is a useful tool for risk assessment, the component structure of livestock trade networks is typically dominated by a so-called giant component (15–19, 24). That is, these networks consist of continents instead of small islands. Consequently, partitioning the network according to components does in general not yield practicable groups for disease control.

In order to find groups in networks that are applicable for disease control, the detection of so-called communities or *modules* has gained considerable attention in veterinary science in the last years (25–30). Modules are similar to components, but allow disjoint groups to be loosely connected. More precisely, a module is a group of nodes that are densely connected to each other, while they have only few connections to other modules.

Finding modules is a promising way to define compartments in networks that can in the best case be isolated from each other in case of an outbreak. Moreover, by now a number of methods is available to find modules even in large networks (31–34). Interestingly, it has been shown that in many cases the modules found for livestock trade networks also show a high spatial clustering, despite the fact that no spatial information is used to infer them [cf. Lentz et al. (26, 27, 29, 30)]. This makes modules potentially interesting for disease control (35, 36). On the other hand, it is well-known that module detection has a resolution limit, i.e., the detected modules cannot be arbitrarily small (34, 37). As an example, the modules found for pig trade in Germany have a scale of federal states (26). Therefore, partitioning such networks into modules is in most cases not feasible for disease control.

Here we use a relatively new method, Bayesian stochastic blockmodeling or simply *stochastic blockmodeling* (SBM) (38), to partition the cattle trade network in Germany into relatively small groups. The SBM method can detect smaller groups than community detection and can even find other structures than densely connected groups of nodes (38). Moreover, stochastic blockmodeling is able to find hierarchically structured groups. Therefore, we can analyze node groups of smaller sizes than those of the classical modules. In order to be applicable to disease control, these groups should (1) show geographical clustering and (2) have a resolution of at least district size, i.e., roughly 30 km (mean district diameter in Germany).

In this work, for the first time we analyze cattle trade in Germany as a complex network. We thereby put a focus on the detection of inherent groups in the network and evaluate the feasibility of different partition methods for disease control. These are community detection for finding modules, a stochastic block model, and a nested stochastic block model with hierarchical structure. Since stochastic blockmodeling is a rather novel method in veterinary applications, we also provide a detailed explanation of the method.

In order to assess the eligibility of modules and block models for animal disease control, we simulate epidemic outbreaks on the network and evaluate different control strategies based on trade restrictions according to different network partitionings. The trade restrictions are realized using targeted edge removal in the network. To compare our results to established methods for disease control, we also simulate trade restrictions based on the geographic closeness of nodes.

This article is organized as follows: We first perform a network analysis of the trade data. Then, we give an explanation of different methods for structure inference, i.e., community detection and the stochastic block model. Finally, we simulate outbreaks on the network and apply different control strategies. The results are integrated into the respective sections.

## 2. Properties of the Network

### 2.1. Data

In this work we analyze an excerpt of the HI-Tier Database (39). The dataset contains cattle movements between farms in Germany from 2010-01-01 until 2014-12-31. Each trade item contains the source farm, target farm and the time of movement. Source and target farms are represented as *nodes* in the network and item as described above is a *trade link*. In this work, trade links are aggregated over time so that two nodes are connected by a directed *edge* whenever there is at least one trade link between them. Overall the network consists of 209,336 nodes and 1,822,373 edges. Using the trade link data without time aggregation yields a temporal network with the same number of nodes, but with 15,416,850 trade links and an observation period of 1,825 days.

### 2.2. Network Analysis

In this section we perform a network analysis of the cattle trade data. A summary of the network measures is given in Table 1. The network is represented by a graph *G* = (*V, E*), where *V* is the set of nodes and *E* is the set of (directed) edges, where each edge connects a node pair.

**Table 1:** Properties of the static network.

**Property**	**Value**
Number of nodes	209,336
Number of edges	1,822,373
Mean degree	17.4
Mean shortest path length	4.4
Diameter	17
GWCC size	0.99
GSCC size	0.69
GIC size	0.21
GOC size	0.07
Path density	0.54

The network can be represented by an adjacency matrix **A**, where an entry (**A**)_*ij*_ = 1, if there is an edge from node *i* to node *j*, and 0 otherwise. The degree *k*_*i*_ of a node *i* is the total number of its neighbors (ingoing and outgoing), i.e., the number of its trade partners. Since we consider a directed network, we also distinguish between in-degree and out-degree for each node.

Indirect connections between nodes, traversing an arbitrary number of edges and no edges or nodes more than once, are called paths. If a path between two nodes *i* and *j* exists, we can write *i* → *j*, and otherwise *i* ↛ *j*. A shortest path between two nodes is a path between them with a minimum number of edges. For the cattle trade network the average shortest path length is 4.4 meaning that a potential disease would take only 4.4 steps on average to infect any node in the network. The maximum shortest path length is called diameter and has a value of 17 for the studied network. Considering the set of all shortest paths between all nodes in the network, the path density ρ_*p*_ is the number of such paths normalized by the number of all possible paths. The path density represents the probability that a randomly chosen node pair is connected in the network. In other words, ρ_*p*_ increases with the overall network connectivity, such that ρ_*p*_ → 1 implies that all node pairs are connected via paths and ρ_*p*_ → 0 implies that the network is fragmented. For our network we have ρ_*p*_ = 0.54.

A concept very related to paths are connected components, which are subsets of nodes *C* ⊂ *V* such that there is a path between all node pairs in this subset. It is a well-known feature of large networks that they possess a so-called giant component, which means that the largest connected component dominates the network and is much larger than the second largest one (9). Giant components form the backbone of complex networks, since they guarantee for the most important feature: connecting nodes. Ignoring the edge directions, the resulting giant component is called giant weakly connected component (GWCC). It contains about 99% of the nodes in our network, i.e., almost all nodes are connected ignoring edge directions. If edge directions are explicitly considered, the resulting giant component is called giant strongly connected component (GSCC), and it only contains nodes that can reach each other on directed paths. The GSCC of the German cattle trade network has a size of 69% of the network nodes. Nodes that are not part of the GSCC, but can reach the latter by a path, form the giant in-component (GIC). This component consist of 21% of the network nodes. In addition, the giant out-component is formed by nodes that can be reached from the GSCC, but do not belong to it. It contains 7% of the network nodes.

## 3. Structure Inference in the Network

In order to efficiently implement disease control in the network based on its topology, the network has to be partitioned into groups that can be easily isolated from each other. It seems natural for this purpose to utilize components as discussed above. However, components are not practicable for disease control due to the existence of the giant component. The latter implies that most nodes belong to a single group and most other groups are irrelevant for disease spread.

On the other hand, the cattle trade network should be comprised of natural substructures—e.g., densely connected node groups or production chains. Merging these subgroups yields the observed network. They are not known from the data set and it is the aim of this section to infer these structures. We first use the well-established method of community detection, and then infer structures using the stochastic block model approach.

### 3.1. Community Detection

A community or module is a set of nodes, where the nodes have significantly more edges within their community than to other communities. Partitioning the network into communities in an optimal way is known to be an intractable problem for large networks (33). One way to obtain an appropriate partitioning of the network into modules is to optimize the modularity function (40, 41)

(1) $$\begin{array}{l} Q=\text{fraction of edges within modules}\\ \;-\text{expected fraction of these edges}. \end{array}$$

The modularity function maps a given partitioning of the network onto a single number. Optimizing Equation (1) means to find the node partition that gives the highest possible value of *Q*. A systematic method to find an optimal partitioning maximizing the modularity function has been proposed in Newman (25). However, the latter method is rather slow and faster methods that perform better even on larger networks have been developed (31, 42). For this work we used the *Infomap* algorithm introduced in Rosvall and Bergstrom (32), which showed a good performance in our network. It can be applied to directed networks and allows for module detection in linear time, that is, the computation time scales linearly with the number of nodes (43).

**Figure 1:**
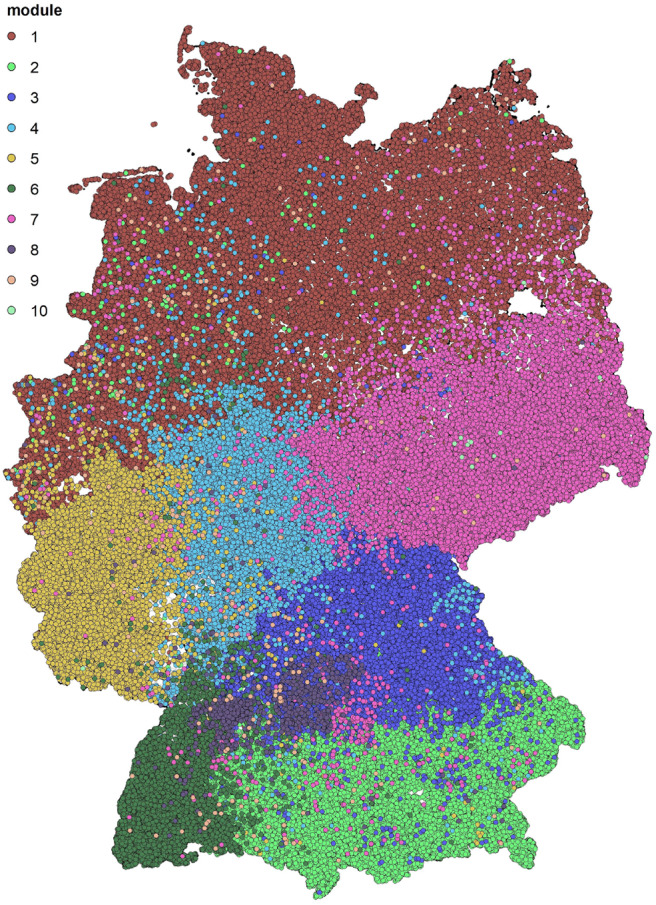
Spatial distribution of the detected groups after modularity maximization. The ten biggest modules are shown. They show high spatial correlation.

After applying the community detection algorithm to the cattle trade dataset, we find modules of sizes between 1 and 73,024. However, 99.89% of nodes are in the 10 largest modules. A map with the 10 largest modules is shown in Figure 1. We note that the detected modules show a high degree of spatial clustering, even though no geographical information has been used for the computation. Some of the found modules reflect borders of federal states (e.g., Rhineland-Palatinate or Hesse). Module 1 represents a whole region of Germany (Northern Germany). In addition, the modules show geographical overlap, which is more pronounced for modules 2 and 3.

### 3.2. Bayesian Stochastic Block Model

**Figure 2:**
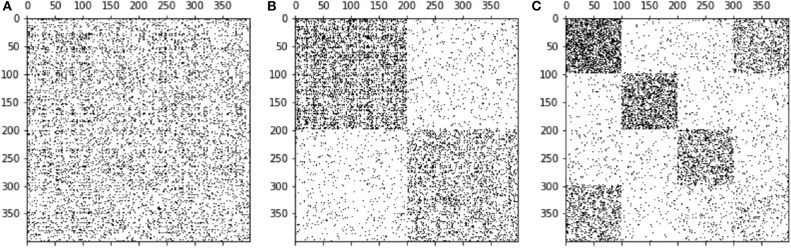
Three different representations of the same adjacency matrix. Through ordering the nodes in various ways we obtain different patterns. Panel **(A)** displays a random order of nodes. Panel **(B)** shows a modular structure, whereas in **(C)** a block structure is observed.

The idea behind a block model of a network is to find groups of nodes belonging together in some way and these groups look like dense blocks in the adjacency matrix **A** (44, 45). These blocks are also called building blocks of the network. As an example, if a network has a community structure as explained in the previous section, the adjacency matrix can be reordered such that nodes of the same module have neighboring indices (say module 1 has nodes 1, …, 100, module 2 has nodes 101, …, 234, and to forth), see Figures 2A,B. Then, the matrix has dense blocks (many edges within communities) along the diagonal, while the rest is almost empty (few edges between different communities). The reason for this shape is that by definition node pairs of the same module have many links, while links to other modules are rare. As opposed to modules, a block model can have a more general structure, e.g., blocks far from the diagonal and no blocks on the latter. An example is shown in Figure 2C. Besides the fact that a block model can resolve more complex network structures than modules, dividing blocks iteratively yields a so-called hierarchical block model. As a consequence, a stochastic block model can resolve relatively small groups in a given network. In fact, it does not suffer from the resolution limit known for community detection (37).

The aim of this section is to infer the underlying block structure from a given network. We now give a brief mathematical sketch of block model inference following Peixoto (45).

At first, we consider the case where the block membership of each node is known in the first place. The network has *B* blocks and the block membership of the nodes is stored in a vector **b**, where the entry *b*_*i*_ is the block membership of node *i*. Furthermore, the number of edges between blocks *r* and *s* is stored in a matrix **E** with entries (**E**)_*rs*_. If we assume that nodes of the same block are statistically indistinguishable, the matrix **E** defines a set of all possible networks with the same topology. Such a set is called *ensemble*. This ensemble is the set of all virtual copies of the network with the same number of edges between blocks, i.e., the same **E**. The number of nodes is also constant.

Within this ensemble, each possible network can be represented by an adjacency matrix **A**. Recalling that the node partition **b** is known, the probability distribution of the possible networks is

(2) $$\begin{array}{l} P\left(\text{A}|\text{b}\right). \end{array}$$

Note that this distribution is a mapping from each virtually possible network to a probability. Due to the large number of possible network configurations, Equation (2) is in general a complicated function. One way to obtain the form of the distribution Equation (2) is to maximize the entropy (or equivalently minimize the information), under certain constraints. The entropy is given by

(3) $$\begin{array}{l} S=-\underset{\text{A}}{\sum }P\left(\text{A}|\text{b}\right)\text{ln}P\left(\text{A}|\text{b}\right), \end{array}$$

and the constraints are first, the matrix **E** containing the edges between groups, and second the normalization of the probability distribution of the networks in the ensemble. Using the method of Lagrange multipliers yields an equation for the desired probability distribution (45).

So far, we have considered the case where the node partitioning was known in the first place. Of course, the problem setting here is exactly the opposite: we have an observed network and want to infer a plausible partition of it. The central idea of the inference algorithm used here is to reverse the distribution Equation 2 using the Bayes formula

(4) $$\begin{array}{l} P\left(\text{b}|\text{A}\right)=\frac{P\left(\text{A}|\text{b}\right)P\left(\text{b}\right)}{P\left(\text{A}\right)}, \end{array}$$

where *P*(**b**|**A**) is the posterior distribution of network partitions given an observed network and *P*(**b**) is the prior distribution, i.e., the distribution of network partitions in the absence of data. If we make no other assumptions, then each partition is equally likely, say *P*(**b**) = 1/*N*_*p*_, where *N*_*p*_ is the number of possible partitions. The term *P*(**A**|**b**) is called evidence and describes the impact of the network data on the prior information, and *P*(**A**) is a normalization constant.

This way we obtain a probability distribution of network partitions (**b**) given an observed network (**A**). The task of finding the optimal partition is equivalent to finding the partition maximizing the posterior distribution, i.e., the left-hand side of Equation (4).

Although formal solutions for this equation exist, these solutions are too complex to find their maxima or even sample from them. Note that, similar to the modularity function Equation (1), Equation (4) maps each possible partition onto a probability. The combinatorial number of such partitions *N*_*p*_ (known as the bell number) is extremely large, and finding the optimal partition of a network is an NP-hard problem, i.e., it is intractable for large networks. For this reason, we utilize a Markov Chain Monte Carlo (MCMC) approach. The idea behind this approach is to start with an arbitrary partition **b**_0_ and change this partition to **b**_1_. This can be realized changing the group membership of a single node. Such a change is accepted, if the resulting partition **b**_1_ increases the posterior distribution (left-hand side of Equation 4). Even if it decreases the latter, it is still accepted with a certain probability. In the long term this procedure results in a random walk in the space of possible partitions and defines a way to sample from the posterior distribution. For the above procedure to converge to the maximum of the distribution, one can slowly reduce the mobility of the random walk, until it remains at the most probable position. This is known as simulated annealing (46).

Although the algorithm above is applicable to the partition problem, it has been shown that convergence can be slow on large networks. For this reason, an optimized MCMC method has been proposed in Peixoto (47). This method is a greedy agglomerative heuristic, i.e., we start with each node being one block and then group nodes together successively. In contrast, in a divisive algorithm, one would start with the whole network as one block and divide until only nodes are left. Divisive algorithms, however, are computationally expensive and agglomerative algorithms are commonly used for large datasets.

The purpose of this section is to give an intuitive understanding of the method of inferring block structures. For a far more comprehensive explanation the reader is referred to Peixoto (38, 45). An implementation of block model inference including the hierarchical version is provided in the software package *graph-tool* (48).

**Figure 3:**
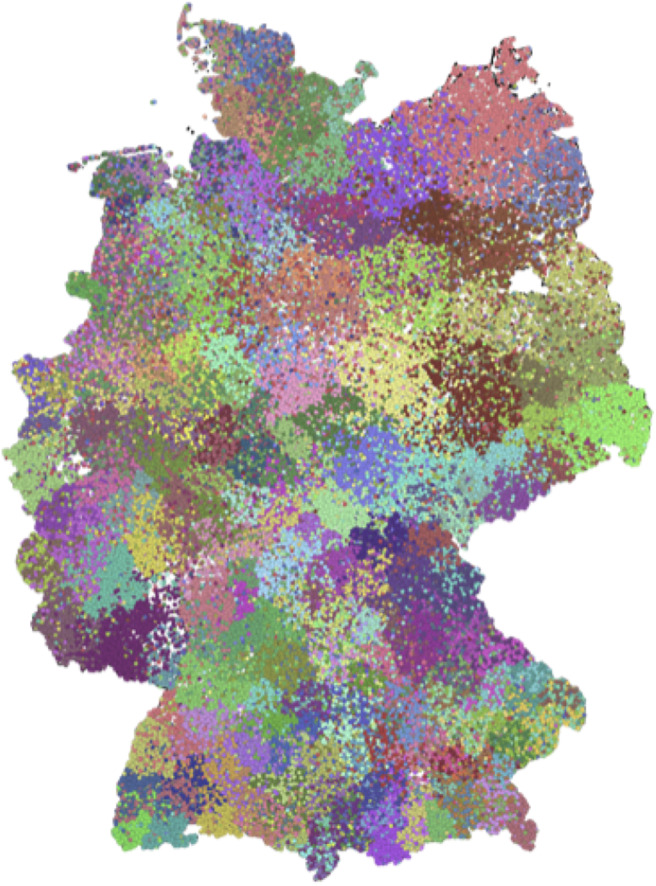
Partition after a block model with 382 blocks. The sizes of blocks roughly correspond to sizes of districts.

Using this software package, we infer a block partition of the German cattle trade network and obtain a partition into 382 blocks. The sizes of blocks are between 1 and 2,887. A map with the block membership of the farms is shown in Figure 3. Similar to the modules, we note that the found blocks show a high degree of spatial clustering, although no geographical information has been used to compute them. Some of the detected blocks reflect borders of districts. Furthermore, some blocks show a geographical overlap.

In addition to this partitioning technique, a block model partition can be inferred for each detected block iteratively (38). The result is called hierarchical or *nested* model. It has the following properties: First, the outcome is a block model where each block is divided into smaller sub-blocks. Second, the resolution of the detected blocks can be increased this way, i.e., blocks found in the lowest hierarchy should have a smaller size than the blocks found using the non-hierarchical method.

**Figure 4:**
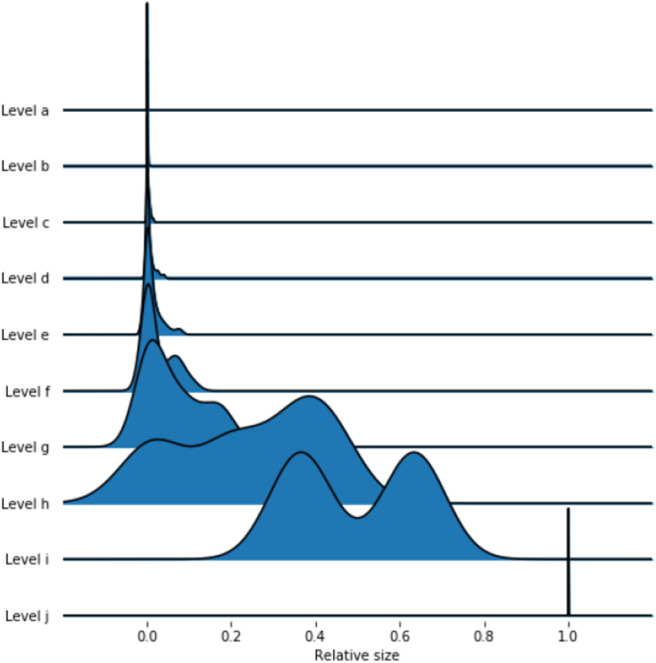
Distributions of block sizes on the different levels. The lower levels have smaller block sizes, while the upper levels are larger.

**Figure 5:**
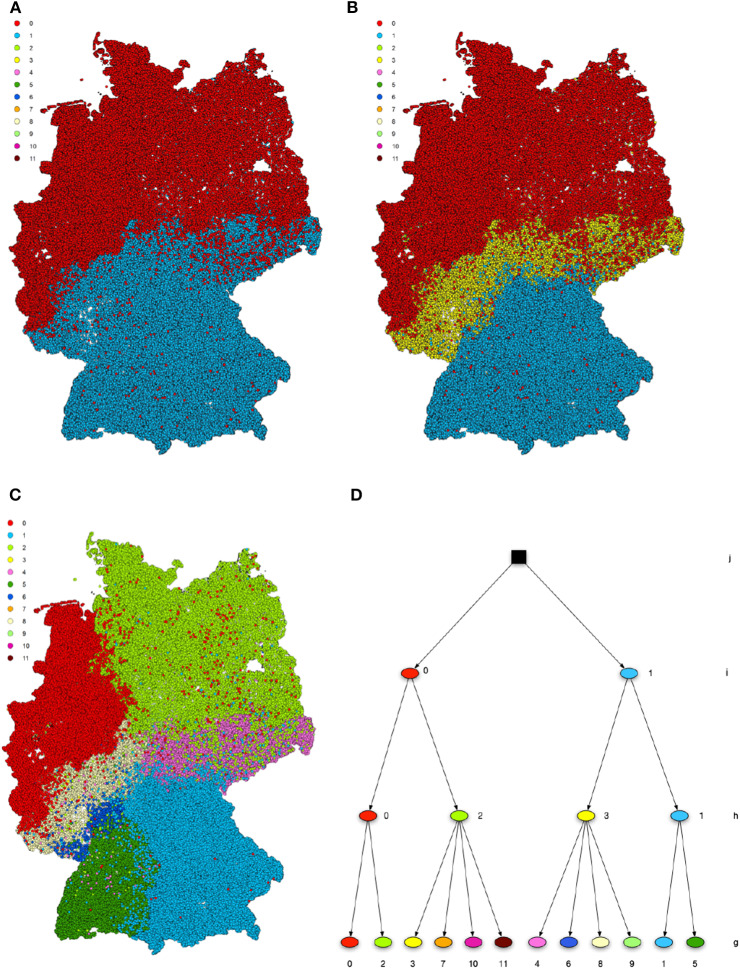
Hierarchical structure after a nested SBM. The levels *i* **(A)**, *h* **(B)**, and *g* **(C)** are geographically shown. The blocks show high spatial correlation on all levels of the hierarchy. Panel **(D)** shows the hierarchy tree.

**Figure 6:**
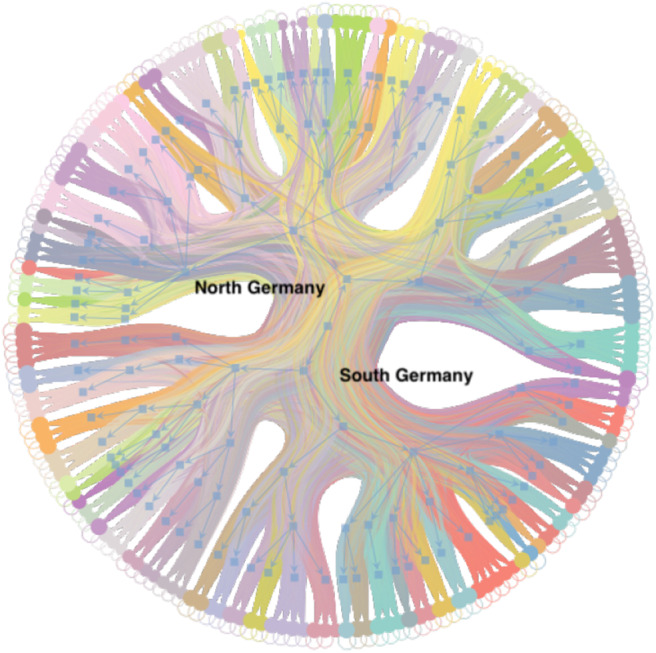
Hierarchy of trade links between blocks of Figure 5. The outer nodes correspond to blocks on level *d*. The blue tree shows the hierarchy and each node of the tree corresponds to a block. The dominant branch in the center separates the northern from the southern German blocks.

Using a *nested* block model to resolve the hierarchical structure of the network, we obtain a hierarchy of ten levels, which are labeled from *a* to *j*. Table 2 contains the number of blocks in each level, while Figure 4 displays the distributions of the block sizes. We consider the highest three levels *g*, *h* and *i* in the hierarchy. Level *j* represents the whole country and is therefore trivial. Figures 5A–C shows the various levels. As above the blocks show a high geographical correlation. The blocks of level *i* strictly divide the country into north (red) and south (blue) Germany. The subjacent level *h* divides the farms by the borders of the regions north (red), central (yellow), and south Germany (blue). Blocks of level *g* (Figure 5C) still reflect larger geographical regions. The north-eastern block (light green points in Figure 5C) contains large parts of federal states like Mecklenburg-Western Pomerania, Schleswig-Holstein, Brandenburg and parts of Lower Saxony. Some blocks reflect borders of federal states (e.g., Bavaria, Baden-Wuerttemberg) (blue points in Figure 5C). The red block spans over several federal states (e.g., Lower Saxony and North Rhine-Westphalia and others). It also shows a large geographical overlap with the light green block. Figure 6 shows the trade links (network edges) between all blocks. The hierarchy is represented by the blue tree, where the root node represents level *j* (whole Germany), its neighbors are level *i* and so on. In the center the dominant branch separating northern from southern Germany is clearly visible. Although the trade structure appears to be complicated, Figure 6 demonstrates that trade links are distributed rather homogeneously between the blocks.

**Table 2:** Block sizes of the hierarchical model.

**Level**	**Number of blocks**
j	1
i	2
h	4
g	12
f	39
e	84
d	180
c	370
b	974
a	2,956

*The block size is measured as the number of blocks in the subjacent level*.

**Figure 7:**
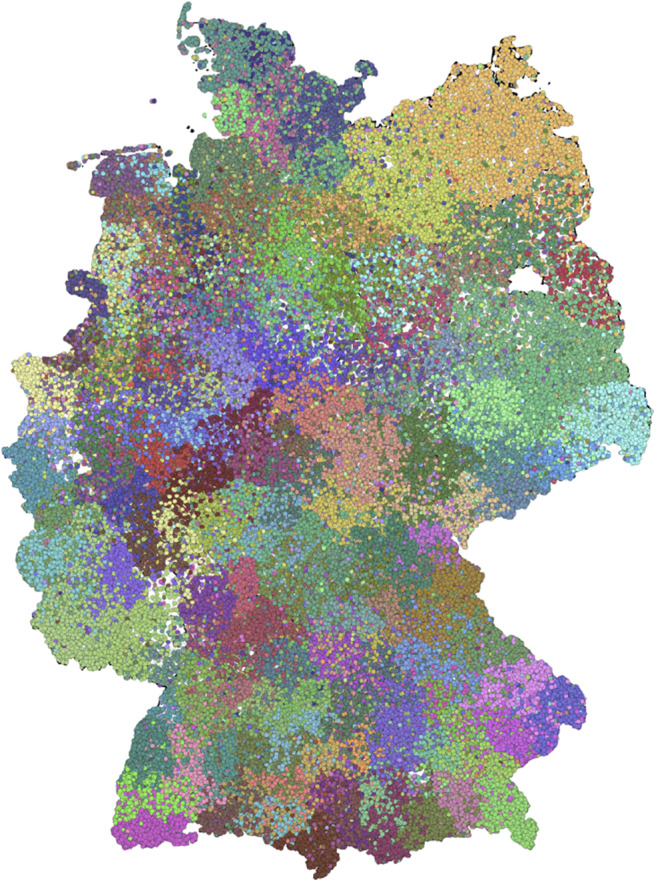
Spatial distribution of level *c* of the nested block model. This level has a block size similar to the non-nested case.

Finally, we check to what extent the nested block model gives a similar result as the non-nested version. Therefore, we choose level *c* (Figure 7), since the number of blocks here is similar to the non-nested version. The figure shows qualitative similarities to the non-nested model (Figure 3). In addition, the SBM on level *c* has similarities with the detected modules (see Supplementary Material).

## 4. Using the Inferred Structures for Disease Control

We evaluate the applicability of the detected modules and block models on disease control by simulating epidemics on the network. Thereby, we utilize different control strategies (see below) based on trade restrictions and compare them to established methods. The established control strategy is based on geographical trade restrictions around a certain radius around the farm where a disease was detected.

**Figure 8:**
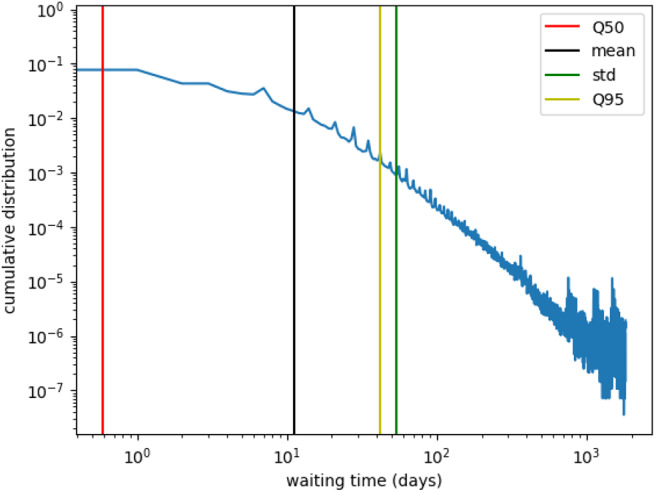
Farm waiting time distribution. The waiting time is the interval in which a farm is not active, e.g., it does not trade. The mean value is roughly 10, i.e., farms trade every 10-th day on average. Therefore, we choose an infection rate β = 0.1.

The infection process is modeled using a so called SI-model, where an infected farm (I) contaminates a susceptible farm (S) upon trade contact with a rate β. Once a farm *i* is infected, it can infect its neighbors, i.e., farms being connected to *i* by a trade link, with rate β. An infected farm stays infectious during the whole simulation. In order to guarantee stable results, we have to choose different initial conditions. Thus, we first sample 10 blocks and then sample 10 nodes out of each block as starting nodes. If we modeled the epidemic process as explained, the disease would infect large fractions of the network within a few steps, since the network is static and all infectious links are permanently active. Therefore, we mimic the temporal nature of trade considering only a fraction of network edges as being present at each step. This fraction can be estimated considering the time span, where a farm does not trade. Out of the data, we observe that nodes are only active every 10 days on average. Figure 8 shows the waiting time distribution (the time span where a farm does not trade) for all nodes of the network. Since the mean value of the waiting time distribution is around 10, we mimic the waiting times using the infection rate, and set β = 0.1.

**Figure 9:**
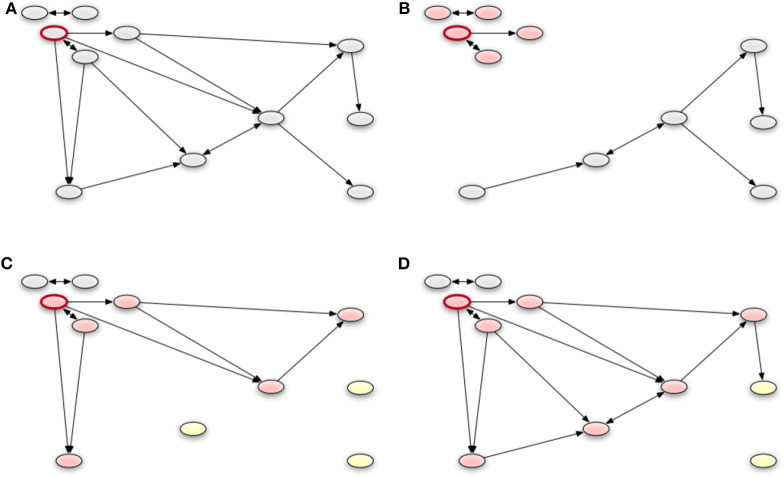
An example of applying the different control strategies of a network. The red framed node represents the index node. We remove all edges of the module or block of the index node that point to other modules or blocks, respectively. Panel **(A)** shows the whole the network. The geographical strategy is represented in **(B)**. Nodes which have a small distance to the index node, build a group (red colored) and there are no edges to nodes with a long distance to the index node (gray). In **(C)**, we see a possible result of the stochastic block model with the blocks. All edges from the block of the index node to the other blocks are removed. A similar result is shown in **(D)**, which presents the groups of modularity optimization. It should be noted that there the group is still similar to the corresponding block but contains more nodes.

In order to assess the performance of different control strategies on the simulated outbreak, we simulate SI dynamics for the different starting nodes and compare the results of the non-nested and nested stochastic block model (level *c*) to the modules found with community detection and the geographical method as explained above. We thereby model disease control measures in terms of trade restrictions. These are realized by removing edges of the trade network according to different schemes. The first control strategy is based on geographical trade restrictions around a 10 km radius of the index farm after detection of the disease (49). Second, we evaluate the applicability of the found modules and blocks by applying trade restrictions at the (edge) boundary of the respective structures. That is, we remove all edges of the module/block of the index farm that point to other modules/blocks, respectively. The different strategies are shown in Figure 9.

Assuming that a disease will spread freely only before it is detected, we remove the trade contacts regarding the infection start node after a detection time *t*_*d*_. We choose *t*_*d*_ = 1 day. This can be considered a best case scenario. Indeed, the detection time depends on the incubation period of the considered disease. However, choosing other values for the detection time does not change the results qualitatively (see Supplementary Material). Due to the fact that the infection process is stochastic, we run the simulation ten times for each starting node.

**Figure 10:**
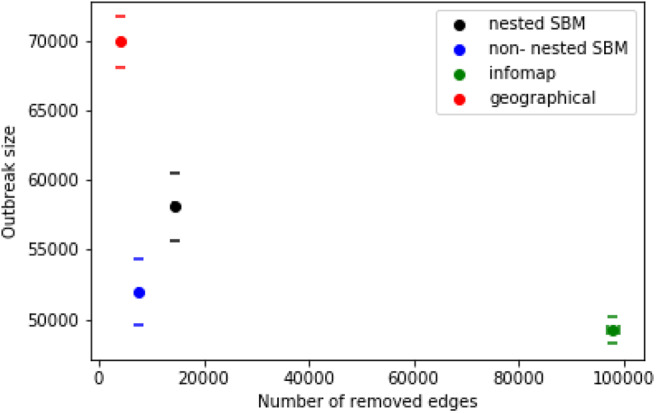
Outbreak size and number of removed edges for different control strategies. Geographic based disease control (red) compared with control based on the block model. Trade restrictions isolating the block of the nested block model of the index node are shown in black, trade restrictions isolating the module of the index node are green. Furthermore the average error of the mean value is shown for each result.

**Figure 11:**
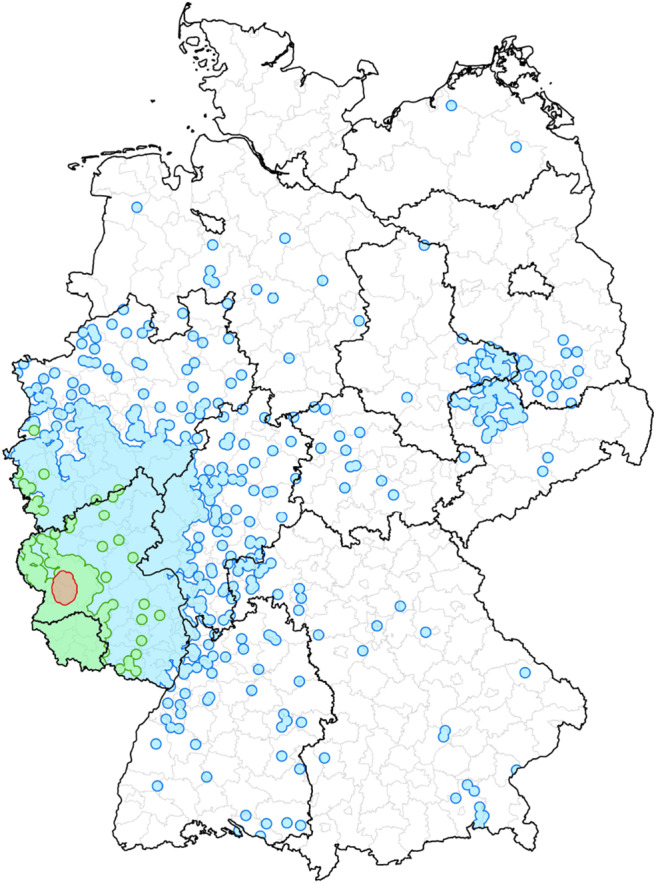
An example to illustrate the different memberships of the same farm. The red area describes the ten kilometer radius around the chosen node and represents the geographical control strategy. Furthermore, the green colored area represents the block affiliation. The module membership of the node is shown as the blue surface.

Figure 10 shows the results of the different control strategies for the nested and non-nested block model. We determine for each strategy the mean values of the outbreak size and the number of edges, which were removed in the simulation. As a consequence of this result the block model leads to a slightly higher number of removed edges in comparison to the geographical method. However, the outbreak size of the block model is significantly smaller. Both strategies, the non-nested block model and the geographical method, perform better than module based trade restrictions in the sense that many edges have to be removed for the latter case and the outbreak sizes are still relatively large. This is due to the fact that the size of the modules is significantly larger than the sizes of the other two groups. Figure 11 shows the sizes of the different groups on a map.

Concerning the nested block model (level *c*), the results are very similar to the non-nested case (see Figure 10). The only difference here is that slightly more edges have to be removed for trade restriction.

An evaluation of different values for the parameters detection time *d*, infection rate β, and radius around the index farm for trade restrictions, is provided in the Supplementary Material. All parameters affect the outbreak sizes and number of removed edges systematically, but do not alter the qualitative results of Figure 10.

In summary, geographically based trade restrictions are superior to module based restrictions. However, stochastic block model based trade restriction outperforms the geographical method yielding smaller outbreaks at a similar number of removed edges.

## 5. Discussion

In this work, we have analyzed the cattle trade network in Germany for the first time. The focus was to evaluate the applicability of different partitions of the network for disease control. As a relatively new method for network partitioning we have used a stochastic block model to infer densely connected farm groups. In contrast to the well-established community detection algorithms, the stochastic block model is capable of detecting relatively small farm groups and can even be used to infer a hierarchical structure. We have found that applying trade restrictions based on a stochastic block model is more efficient for disease control than geographical, or module based trade restrictions.

Disease control has been implemented in this work as trade restrictions, and the disease spread follows a relatively simple model, i.e., the SI-model. Even though this model oversimplifies the course of most relevant diseases, the infection mechanism in the beginning of the outbreak can be approximated by an SI-process in most cases. We provide a comparison between the SI-model and the SIR-model (susceptible - infected - recovered), where farms are removed from the infection process after a certain period, in the Supplementary Material. The difference between the two models is marginal for detection times less than 14 days.

In contrast to geographically based trade restrictions network based restrictions are not guaranteed to be constant over time since trade patterns might change (15, 18, 24, 50, 51). In applications trade boundaries could simply be computed using current trade data so that temporal constancy plays a minor role. Moreover, it is plausible that particularly larger node groups show only small fluctuations over time (24).

As we have demonstrated, modules as well as blocks show a high degree of spatial clustering. Even though this property is also used in the geographical approach, the trade data offers still another way of node partition: the underlying production chains. A stochastic block model should in principle be capable of finding such structures as well. For example, functional blocks in the world trade network have been found in Reichardt and White (52), where the authors could resolve the role of different countries in global economy. However, this requires a relatively complex null model (mathematically speaking in the form of constraints in the optimization) in the inference algorithm. It would be interesting for future work to validate different null models in order to resolve production chains. If the production chains were known, we could implement economically efficient trade restrictions allowing for redirecting trade channels in the case of an outbreak.

As our results show, the application of a hierarchical block model on cattle trade data seems to be a promising approach for applications in livestock disease control. Moreover, decoupling trade restrictions from geographical neighborhood protects the neighborhood of the index farm from being considered false positive, and thus might contribute to animal welfare. However, these statements only holds if we neglect current legislation for disease control, and it is of course beyond the scope of this paper to change legislation. Nevertheless, the strategy for trade restriction presented here is technically feasible, i.e., only low computational power is needed and block structures could be inferred on the fly, or at least on a regular basis, in order to have on-time trade groups. We therefore believe that groups in trade data are useful in application and could improve disease control.

## Data Availability Statement

The datasets analyzed in this article are not publicly available. Requests to access the datasets should be directed to hartmut.lentz@fli.de.

## Author Contributions

LB performed the analyses. LB and HL wrote the manuscript. HL and MF designed the study.

## Conflict of Interest

The authors declare that the research was conducted in the absence of any commercial or financial relationships that could be construed as a potential conflict of interest.
