# Corrigendum: Hierarchical Structures in Livestock Trade Networks—A Stochastic Block Model of the German Cattle Trade Network

**DOI:** 10.3389/fvets.2020.00456

**Published:** 2020-07-30

**Authors:** Laura Brzoska, Mareike Fischer, Hartmut H. K. Lentz

**Affiliations:** ^1^Institute of Mathematics and Computer Science, University of Greifswald, Greifswald, Germany; ^2^Institute of Epidemiology, Friedrich-Loeffler-Institut, Greifswald-Insel Riems, Greifswald, Germany

**Keywords:** network analysis, epidemic model, cattle trade, Germany, modularity, stochastic block model

In the published article, there was an error regarding the affiliation(s) for Laura Brzoska. As well as having affiliation 1, they should also have affiliation 2.

The authors apologize for this error and state that this does not change the scientific conclusions of the article in any way. The original article has been updated.

