# Supplementary material for: Hierarchical Structures in Livestock Trade Networks—A Stochastic Block Model of the German Cattle Trade Network
Source: Front Vet Sci. 2020 May 27;7:281. doi: 10.3389/fvets.2020.00281 (PMC7266987; doi:10.3389/fvets.2020.00281)
Supplement: Supplementary file 1 [file Presentation_1.pdf]

![frontiers]

Supplementary Material – Hierarchical Structures in Livestock Trade Networks

1 BLOCK MODEL VS. MODULES

Figure S1 shows the mixing matrices of the network – i.e. the number of edges from group i (rows) to group j (columns) – for the different detection algorithms. Figure S1 A shows the mixing matrix after community detection. Only the ten largest modules are shown. The matrix is clearly dominated by groups along the main diagonal, as expected for community detection. Modularity is $Q_{\text{modules}} = 0.58$.

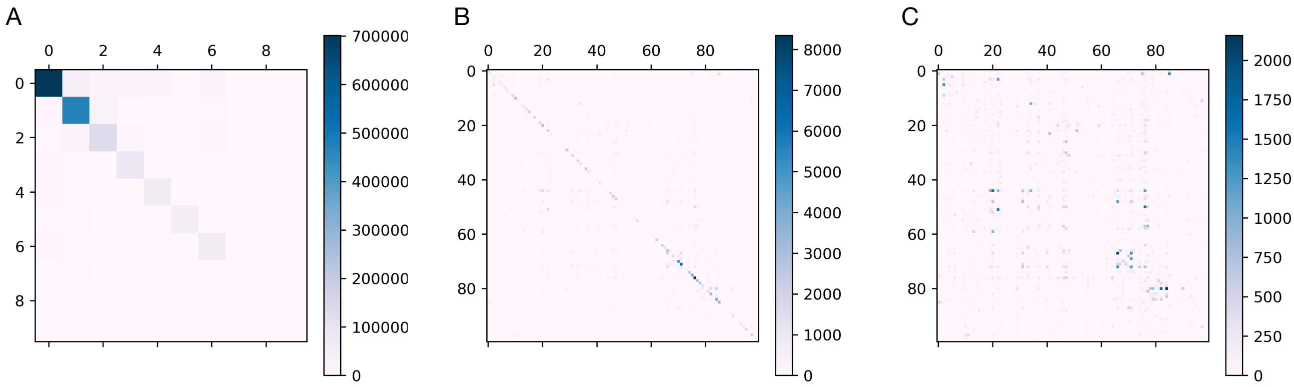

Figure S1. Mixing matrices of the network after community detection (A), and for the hierarchical stochastic block model (B). Only the ten largest modules and the first 100 blocks are shown. Removing blocks along the main diagonal of (B) reveals additional structures (C).

Figure S1 B shows the mixing matrix of the hierarchical block model on level c (only the first 100 blocks are shown for clarity). Still, the main diagonal is clearly visible as it was the case for module detection and thus modules are structures also present in the stochastic block model. The modularity for the hierarchical block model (level c) is $Q_{\text{SBM}} = 0.32$, and that of the non-nested model is similar. Moreover, other mixing patterns are present beside the main diagonal. Figure S1 C shows the same matrix as Figure S1 A, but without the main diagonal blocks.

2 IMPACT OF PARAMETERS ON THE OUTBREAK MODEL

In order to test the robustness of the parameters on the outbreak sizes and number of removed edges, we investigate the model outcomes for different parameter values.

2.1 Impact of radius for geographical trade restrictions

Figure S2 shows the results of the different control strategies in comparison to a geographical restriction with a 3 km radius. Outbreak size and number of removed edges appear to be robust against variable radii.

2.2 Impact of detection time and transmission probability

As a typical example of viral diseases, foot-and-mouth-disease has an incubation period between 2 and 14 days (OiE, 2020). If we assume that clinical signs appear after this period, we equalize the incubation period with the detection time t_d. Figure S3 shows the results for different values of t_d, where crosses

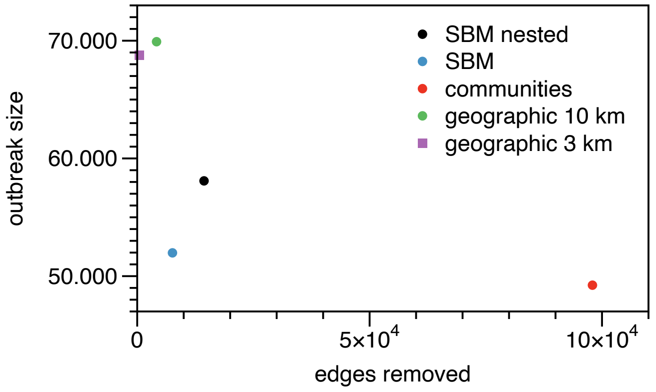

Figure S2. Impact of the radius. The points display the results of the main paper. Whereas the purple square shows the result of geographical trade restriction with a radius of 3 km around the index farm.

stand for $t_d = 1$ day, routes for $t_d = 7$ days, and circles for $t_d = 14$ days. The different colors indicate the different trade restriction strategies.

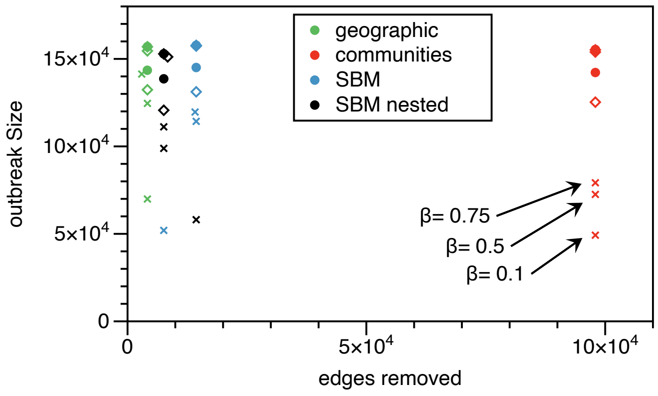

Figure S3. Impact of the detection time d and transmission probability β on the results of the main text. Parameters: Crosses: $t_d = 1$ day, routes: $t_d = 7$ days, circles: $t_d = 1$ days. Increasing β increases the outbreak size for all scenarios.

In addition, we vary the value of the transmission probability β. This has a systematic impact on the result, i.e. small values of β result in small outbreaks, whereas larger values result in larger outbreaks. This is exemplarily shown for the community based trade restriction for $t_d = 1$ day. The tendency is the same for all other strategies and parameters.

In summary, increasing the detection time shifts the outbreaks to larger values, but does not alter the behavior of the results qualitatively. The same is true for different values of β. Consequently, the results of the main text are not (qualitatively) altered when other parameter values are considered.

3 SI-MODEL VS. SIR-MODEL

Figure S4 shows the dynamics of the SIR (susceptible, infected, recovered) and the SI-model. The recovery probability per time step is $\mu = 0.05$. The relevant time period for this paper is determined by the detection

time, which is in all cases less than 14 days. Consequently, the differences between the model outcomes of the SI and SIR models are marginal.

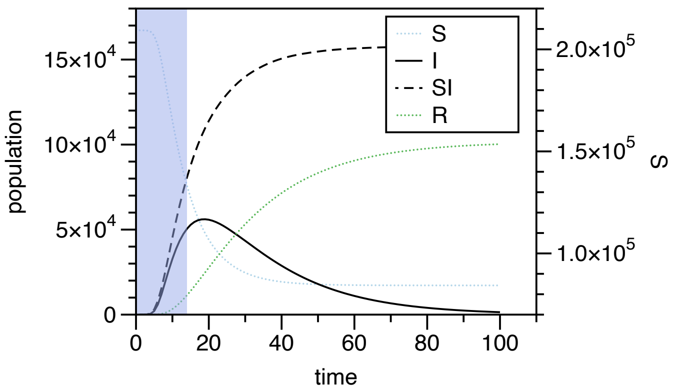

Figure S4. Comparison of a SI-model and SIR-model. The black line shows the number of infected nodes in the SIR-model. Infected nodes in the SI-model are shown as a black dashed line. In the beginning of the infection process (the first 14 days are highlighted in blue) the difference between both models is small.

REFERENCES

[Dataset] OiE (2020). https://www.oie.int/en/animal-health-in-the-world/animal-diseases/foot-and-mouth-disease/
